# A new paradigm for addictions

**DOI:** 10.1093/alcalc/agad027

**Published:** 2023-04-21

**Authors:** Kari Poikolainen

**Affiliations:** Department of Public Health, University of Helsinki, Finland

**Keywords:** addiction, dependence, concept, hyperbolic discounting, paradigm, pleasure, risk factors

## Abstract

**Aim:**

To suggest a new paradigm for addictions.

**Methods:**

Consideration of relevant research findings and thought experiments.

**Results:**

Common mental motors leading to addictions are pleasure-seeking and hyperbolic discounting. The important point of the latter is that given two choices of future rewards, commonly one initially prefers the larger one available after a longer waiting time but despite this the smaller and sooner reward will be chosen when it becomes available. These are general biological properties, found at least in human beings, the rat, and the pigeon. If this continues it may create an unconscious habit, difficult to change. Several other risk factors for addictions are known, notably both externalizing and internalizing mental problems. Predisposing factors are likely to interact.

**Conclusions:**

The above suggests a new paradigm for addictions. Pleasure provides temptations, hyperbolic discounting weakens the will. Habits emerge. Addictions seem to be a group of problems of its own kind, not diseases, because diseases do not bring about pleasure, and are not sought for pleasure.

## Introduction

Addictions to gambling, sex, eating, abuse of power and pleasure-inducing chemicals (like alcohol, drugs, and tobacco) are commonly known. Other types of addictions have been suggested. We seem to live in an age of addictions.

Addictions have been thought to be a sin, craving, bad habit, lack of self-control, or dependence. There is a vast literature on models (also called paradigms or theories) aiming to understand and explain addictions. A recent Google Scholar search yielded ⁓32 900 reviews on theories of addictions and addictive behaviors. Various models have been both endorsed and criticized (for example see Davies, 2018 and Heilig *et al*., 2021). While a thorough review of this literature is beyond the scope of the present essay, three major models are briefly described below.

The chronic brain disease model assumes that there are essential changes in neural circuits involved in reward, stress, and self-control, explained by abnormalities in chemical processes in the brain. Risk of addiction is thought to depend on variations in both genetic and early environmental factors that “hijack” neural pathways (NIDA, 2018; Heilig *et al*., 2021). The motivational model sees addiction as a fuzzy collection of symptoms rather than a disease. The symptoms are thought to be brought about by motivational imbalances (West and Brown, 2013). The rational choice model assumes that addiction is normal behavior due to giving more weight to utilities (rewards) of addictive behavior than alternative options (Becker and Murphy, 1988). All the above models tap on some features of addiction, but no one can explain them all. The present essay tries to take one step further by sketching an alternative paradigm. It is based on the common behavioral motivations of pleasure-seeking and goal-setting of rewards.

## Hyperbolic discounting

The life-supporting pleasurable activities provide a more immediate reward sooner than learning social and occupational skills, but the reward is usually smaller. When one chooses between a sooner and smaller or a larger and later reward, a rational choice is to always prefer the latter. In this case the functions showing how the current, discounted value of the two choices change over time from the present to the time to receive the reward are exponential; indicating that one sticks to the original preference all the time. Alas, this is less common than changing the preference to the smaller and sooner reward when the latter appears to be immediately available. Such discount functions are hyperbolic and the cross-point indicates the time of the preference change. This has been observed in human beings, the pigeon, and the rat. The reward in these studies has been either money or commodities, real or fictional (Oliveira *et al*., 2014). This helps to understand the difficulty to keep promises of cutting down excessive behaviors.

The point of change varies between individuals and situations. Ascertaining this point may help to identify persons who are at risk of addiction, to ascertain potential risk factors, estimate the severity of addiction and improvement in treatment (Bickel *et al*., 2014).

Change of goal preference is common but not universal (Story *et al*., 2014). Some prefer the larger and longer goal all the time, others may prefer the opposite. The latter consist of ⁓3–10% of population with respect to the less good health-related goals in various studies (Story *et al*., 2014). There is some correlation between money, smoking, and alcohol intake discounting, although not strong. The correlations are stronger between money rewards and illicit drugs.

Discounting patterns seem to show some stability. However, the patterns can change over time. Self-control of illicit drug abuse has been found to improve after memory exercises (Story *et al*., 2014).

Actual behavior is influenced, in addition to discounting patterns, by common environment, specific situation, chance, and other unknown factors. Conscious decisions are based on weighing the available options according to goal-directed models. When these are repeated the behavior becomes automatic, just like subconsciously learned behaviors (Story *et al*., 2014). Habits have been formed.

## Pleasure and information processing in the BRAIN

From the evolutionary perspective it makes sense that life-supporting activities like eating, drinking, procreation, caring for offspring, and interpersonal relationships are pleasurable. Dopamine is central in these activities. Several neural pathways transfer incoming data to the ventral tegmental area in the brain. Dopamine release then results in information transfer to brain areas responsible for emotions, executive functions, and memory. Generally, the role of the dopamine neurons in relation to reward is to assess the difference between an anticipated reward and the experienced reward. This assessment would then serve as the basis of experiential learning (Arias-Carrión *et al*., 2010; Kalant, 2010).

In most contemporary theories of emotion, the processes of desire, happiness, and pleasure are separable yet affect each other. These theories are in line with Sigmund Freud’s concept of erotic pre-pleasure that makes humans to search for ways of satisfaction (Gay, 1995). This search increases mental tension. Satisfaction arouses the desire to get more of the same. Hence, the aim is always to get more satisfaction. There is no end to this, no final goal. To quote Cecil Rhodes: “I would annex the planets if I could; I often think of that.”

Desire reflects a focused interest in a goal and the drive to reach it. Happiness is an emotional state linked to the feeling of progress toward the goal. The experience is called “flow” by some. Pleasure is a positive feeling when the goal has been reached. In the brain, dopamine transmission influences each of the above processes. Findings suggest that dopamine plays a major role in wanting the addictive behavior, and a substantial role in supporting happiness when seeking and preparing for this behavior.

Dopamine seems to influence the pleasure resulting from the addictive activity to a lesser extent (Leyton, 2010). Dopamine levels have been found in many studies to be increased when eating tasty food, winning (and even losing) money when gambling, and using alcohol, tobacco, amphetamine, and cocaine (Leyton, 2010; Ainslie, 2013). A few small studies have found similar changes in relation to beauty, sex, pornography, and romantic love. The levels return to normal when the positive emotions wane. To sum up, studies have shown that dopamine-dependent brain circuits are activated in many addictions, both those due to drugs and those not.

The brain disease view assumes implicitly that there are flaws in the functioning of the dopamine-dependent neural circuits brought about by heavy use of mind-altering substances. These are associated with the rewarding effects of drugs abused (Heilig *et al*., 2021). However, no clear flaw has been found that could ascertain with reasonable accuracy the presence of an addiction and point out how it could be remedied.

It may be impossible to prove without any doubt that the basic neurobiological mechanisms of reward are similar in addicted and non-addicted individuals, but many findings support this view. Dopamine activity seems to be behind both the distress-alleviating and pleasure-inducing placebo effects (Petrovic, 2009). Increased dopamine activity is related to listening to pleasurable music (Zatorre, 2015). Human neuroimaging studies indicate that surprisingly similar circuitry is activated by quite diverse pleasures, suggesting a common neural currency shared by all (Berridge and Kringelbach, 2015). Thus, it seems probable that there is no pivotal difference in the dopamine-dependent brain functions and that the quest for pleasure is a normal motivational force.

## Possible risk factors

Many possible risk factors or correlates for addictions have been found. The magnitude and the strength of evidence varies between studies. The occurrence of addictions is higher in lower social groups compared with the higher ones. Causes are likely to be many. A rich person may become poor because of an addictive lifestyle. Addicts may be less intelligent, less conscientious, less stress-resistant, and less educated than the rest. They may have more often externalizing disorders (like intermittent explosive, antisocial personality, and attention-deficit hyperactivity disorders) or internalizing disorders (like anxiety and depression disorders). For example, in a Swedish representative sample of 217 000 persons, 32% of alcohol addicts had a background of externalizing, 23% internalizing type of problems, while the rest were found to show but minimal evidence of psychopathology (Kendler *et al*., 2022).

Hyperbolic discounting, mental health problems, and addictions are related to each other. For example, hyperbolic discounting is more common among impulsive cocaine addicts than among controls (Coffey *et al*., 2003), and among depressive patients (Pulcu *et al*., 2014). The cause-and-effect paths are largely unknown. However, discounting pattern may often be the primary cause. Already among 4-year-old children there are differences in the ability resist the temptation to take a smaller marshmallow reward if waiting yields a larger one. This has been found to associated with later competence and academic achievement (Casey *et al*., 2011).

## Severity and LIFE trajectories

Addiction is a fuzzy concept and thus undeniable diagnosis is not possible. Certainly, explicit criteria can and have been set, but there can be much variation in applying these in practice. Nevertheless, some arbitrary line of distinction is needed if the decision to treat or not has to be made. This may depend much on the training of the diagnostician, attitude, and self-report of the prospective patient and the social and cultural values present, and this is one probable reason for wide differences in relative risk and occurrence estimates. There seem to be several degrees in the severity of addiction. The American taxonomy Diagnostic and Statistical Manual of Mental Disorders (DSM-5) provides three degrees for alcohol use disorder. The severity may change from zero to high in an addict over his or her life history. Little is known about such long-term trajectories. Perhaps mild- and short-term episodes of addictions are common but go unnoticed.

## Discussion

The new paradigm suggests that pleasurable activities provide the temptation and hyperbolic discounting of rewards the weakness of the will, leading to addictions. This seems to be the primary cause, necessary but not sufficient. Mental disorders and other individual level factors then increase the risk as well as social and environmental factors. Moreover, the antecedents of addictions may interact. For example, hyperbolic discounting differs between impulsive and non-impulsive alcohol addicts (Westman *et al*., 2017). The roads to addictions are long, complex, and vary between individuals.

The present paradigm subsumes the rational choice model. At some time in their life course future addicts may consider their addictive behavior rational even if the general public sees their choice irrational. The present paradigm is more parsimonious than the overarching motivational model, since the focus is on the biological findings operating at the behavioral level instead of underlying chemistry. It is less complex than the chronic disease model. The latter aims to clarify differences in neural processes between addicts and non-addicts at the biochemical level. The present paradigm differs from the above models by emphasizing that succumbing to pleasurable temptations because of hyperbolic discounting in choice situations is likely to lead into habit formation.

Both pleasurable rewards and hyperbolic discounting are general mental motors of behavior. Neurobiological research supports the view that there are no important differences in the neural processes between those addicted and the rest. Harold Kalant (2010) has concluded “… it is inherently impossible to explain addiction by pursuing only the analytical study of drug interactions with the nervous system at ever-finer levels of molecular structure and function.” Addictions seem to be extremes of normal variation in biological functions. In addictions, the mental motors of behavior seem to be highly tuned and over revved. The reasons for this are largely unknown. The multitude of risk factors, their usually low relative risk estimates, and the variation in life trajectories of addictive behaviors, suggest that we have here a heterogeneous bunch of problems that is hard to tackle.

Addictions seem to be a group of problems of their own kind, not diseases, or what is commonly thought of as disease. Diseases do not bring about pleasure. Moreover, diseases are not an object of desire. A diabetic cannot get rid of the disease just by wishing it away, but an addict can exercise self-control (Davies, 2018). Addicts have free will, although sometimes a weak one. Recall the prayer of Saint Augustine: “Grant me chastity and continence, but not yet.”

While addictions are not diseases, it does not follow that the medical profession should not try to treat addicts, if they can. (The profession can assist problems in giving birth, cosmetic deformities and other problems that are not diseases).

The above new paradigm seems to agree better with what is known about addictions than other models. Hopefully, it helps to understand the nature of addictions in a way that may also open new paths to p revention and treatment.
